# Knockdown of PGC1α suppresses dysplastic oral keratinocytes proliferation through reprogramming energy metabolism

**DOI:** 10.1038/s41368-023-00242-3

**Published:** 2023-09-04

**Authors:** Yunkun Liu, Nengwen Huang, Xianghe Qiao, Zhiyu Gu, Yongzhi Wu, Jinjin Li, Chengzhou Wu, Bo Li, Longjiang Li

**Affiliations:** 1 State Key Laboratory of Oral Diseases & National Center for Stomatology & National Clinical Research Center for Oral Diseases & Department of Head and Neck Oncology, West China Hospital of Stomatology, Sichuan University, Chengdu, China; 2 Department of Preventive and Pediatric Dentistry, Hospital of Stomatology, Zunyi Medical University, Zunyi, China; 3 State Key Laboratory of Oral Diseases & National Center for Stomatology & National Clinical Research Center for Oral Diseases & Department of Orthodontics, West China Hospital of Stomatology, Sichuan University, Chengdu, China

**Keywords:** Oral cancer detection, Oral cancer

## Abstract

Oral potentially malignant disorders (OPMDs) are precursors of oral squamous cell carcinoma (OSCC). Deregulated cellular energy metabolism is a critical hallmark of cancer cells. Peroxisome proliferator-activated receptor-gamma coactivator-1 alpha (PGC1α) plays vital role in mitochondrial energy metabolism. However, the molecular mechanism of PGC1α on OPMDs progression is less unclear. Therefore, we investigated the effects of knockdown PGC1α on human dysplastic oral keratinocytes (DOKs) comprehensively, including cell proliferation, cell cycle, apoptosis, xenograft tumor, mitochondrial DNA (mtDNA), mitochondrial electron transport chain complexes (ETC), reactive oxygen species (ROS), oxygen consumption rate (OCR), extracellular acidification rate (ECAR), and glucose uptake. We found that knockdown PGC1α significantly inhibited the proliferation of DOKs in vitro and tumor growth in vivo, induced S-phase arrest, and suppressed PI3K/Akt signaling pathway without affecting cell apoptosis. Mechanistically, downregulated of PGC1α decreased mtDNA, ETC, and OCR, while enhancing ROS, glucose uptake, ECAR, and glycolysis by regulating lactate dehydrogenase A (LDHA). Moreover, SR18292 (an inhibitor of PGC1α) induced oxidative phosphorylation dysfunction of DOKs and declined DOK xenograft tumor progression. Thus, our work suggests that PGC1α plays a crucial role in cell proliferation by reprograming energy metabolism and interfering with energy metabolism, acting as a potential therapeutic target for OPMDs.

## Introduction

Deregulated cellular energy and enabling replicative immortality are critical hallmarks of cancer cells.^1^ By regulating the production and release of growth-promoting signals, cancer cells evolve various mechanisms to reprogram their energy metabolism pathways to fuel and sustain unlimited cell proliferation.^2^ Aerobic glycolysis is the most characterized metabolic phenotype of cancer cells, which ferments glucose into lactate even if there is sufficient oxygen to support mitochondrial oxidative phosphorylation (OXPHOS).^3^ Although OXPHOS produces 38 molecules of adenosine-three-phosphate (ATP) via the complete oxidation of one glucose molecule, cancer cells have 19 times higher glucose uptake to provide the same energy as well-oxygenated cells and biosynthetic precursors for supporting anabolic reactions during the rapid proliferation.^4–7^ Studies concerning the hallmarks of cancer helped us better understand the pathogenic mechanisms involved in the development of cancer, including oral squamous cell carcinoma (OSCC), which have provided great prospects for developing cancer treatments by targeting metabolic pathways of cancer cells.^8^

The peroxisome proliferator-activated receptor-gamma coactivators 1 alpha (PGC1α) belongs to the PGC1 family and has received the most extensive study because of its critical involvement in mitochondrial biogenesis network, energy expenditure, and molecular transcription regulation.^9–12^ Besides, it plays crucial roles in regulating of adaptive thermogenesis, mitochondrial biogenesis, oxidative phosphorylation, muscle fiber-type switching, gluconeogenesis, and clock gene expression.^13,14^ In recent years, it has been described that PGC1α played an essential role in pro- or anti-tumorigenic effects on melanoma, breast cancer, endometrial cancer, prostate cancer, thyroid cancer, and hepatocellular carcinoma, affects tumors progression, metastasis, metabolic state, and the response to treatments.^15–17^ Researchers found that PGC1α suppresses hepatocellular carcinoma metastasis by inhibiting aerobic glycolysis via regulating the WNT/β-catenin/PDK1 axis.^18^ In contrast, PGC1α is downregulated in prostate cancer and suppresses its progression and metastasis by activating an ERRα-dependent transcriptional program.^19^ Although low levels of PGC1α expression and mitochondrial copy numbers were observed in OSCC, the biological function and molecular mechanism of PGC1α in the progression and energy metabolism of OSCC remain elusive.^20^

Oral potentially malignant disorders (OPMDs) are precursors of OSCC, with a worldwide prevalence of 4.47% and a mean risk of malignant transformation rate of 12%.^21,22^ Most of OPMDs are asymptomatic in early stages, usually ignored, and prone to becoming malignancy. Therefore, early detection and interference of OPMDs in dental clinics can significantly improve treatment outcomes and prognosis, thus saving patients’ lives. For example, high-risk oral leukoplakia (OLK) patients should receive more frequent follow-up and more aggressive treatment to avoid carcinogenesis, while low-risk patients need more conservative management.^23,24^ Even with annual monitoring, OPMDs patients spend less than oral cancer patients.^25^ However, there is no effective and quantitative method in common use for OPMDs malignant transformation risk assessment, and heterogeneity in OPMDs influences treatment strategy and prognosis.^22,26,27^ Therefore, investigating the molecular mechanisms underlying OPMDs’ progression is urgently needed for developing treatment options and terminating the progression of the disease, thus improving the prognosis for OPMDs patients.

Decreased PGC1α in tumors may be responsible for enhancing aerobic glycolysis. However, whether PGC1α is involved in OPMDs is not known. Therefore, we aimed to uncover the effects of PGC1α in OPMDs and to investigate the underlying mechanism from an energy metabolism standpoint. This knowledge is critical in determining whether the intervention of the energy metabolism pathway is necessary to prevent the malignant transformation of OPMDs.

## Results

### Knockdown of PGC1α inhibits proliferation of DOKs and tumorigenesis in vivo

**Fig. 1:**
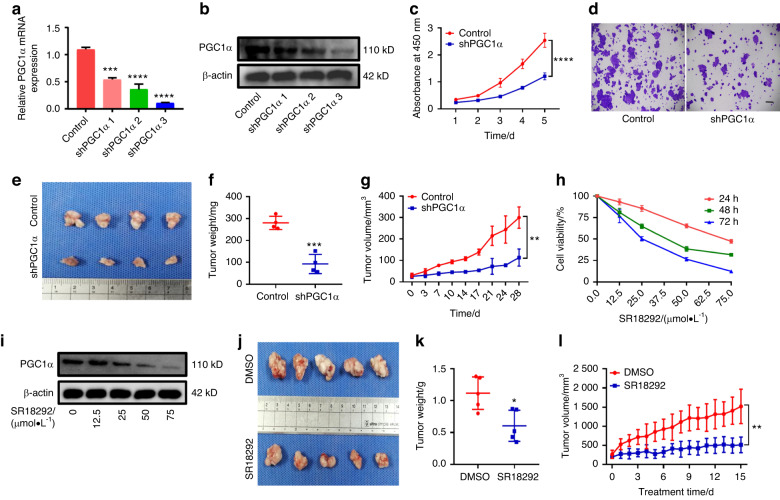
PGC1α knockdown suppressed DOKs proliferation. **a**, **b** The expression of PGC1α was detected by RT-PCR (**a**) or western blot (**b**) in DOKs after being infected with PGC1α knockdown or control lentivirus. **c** CCK-8 assays detected growth curves of DOKs. **d** A colony-formation assay was performed to measure the number of colonies in the control and shPGC1α DOK cells, the scale bar indicates 200 μm. **e** Xenograft tumors were shown. **f** Weight of xenograft tumors. **g** Growth curves of xenograft tumors in control and shPGC1α DOKs were shown during the 4 weeks. **h** CCK-8 assay in DOKs treated with SR18292 (0–75 μmol·L^−1^) for 24, 48, or 72 h. **i** Western blot analyses of PGC1α expression in DOKs treated with SR18292 (0–75 μmol·L^−1^) for 48 h. **j** SR18292 inhibits the growth of the DOK xenograft tumors model. **k** Weights of xenograft tumors mice of each group. **l** Growth curves of xenograft tumors from DOK tumor-bearing mice injected with 5% DMSO or SR18292 daily for 15 days. *P < 0.05, **P < 0.01, ***P < 0.001, ****P < 0.000 1

As a potent regulator of mitochondrial function, we hypothesized that suppression of PGC1α will affect the biological behavior of DOKs. First, we established a stable PGC1α knockdown DOK cell line, and knockdown efficiency was verified by RT-PCR and western blot (Fig. 1a, b). Then, we investigated whether PGC1α knockdown influenced cell growth. As a result, PGC1α knockdown significantly inhibits the proliferation of DOKs (Fig. 1c). Consistently, the clone formation assay showed that PGC1α knockdown cells formed fewer and smaller clones (Fig. 1d). To further determine the effects of PGC1α knockdown on tumorigenesis in vivo, control and shPGC1α DOKs were subcutaneously injected into nude mice. As shown in Fig. 1e–g, mouse xenograft models displayed that the shPGC1α group had smaller tumor volumes, slower tumor growth, and lower weight of tumor xenografts than the control group, but without affecting the weight of mice in the two groups (Supplementary Fig. S1a). Altogether, these results indicated that suppressing the expression of PGC1α inhibits DOKs proliferation.

Next, we assessed the cytotoxic effects of SR18292 (an inhibitor of PGC1α) on DOKs. CCK-8 results showed that SR18292 inhibited the proliferation rates of DOKs in a dose- and time-dependent manner (Fig. 1h). And SR18292 treatment progressively reduced PGC1α levels (Fig. 1i). Whether the anti-tumor efficacy observed in vitro could induce similar responses in vivo. A xenograft tumor model was established by subcutaneously injecting DOKs into BALB/C nude mice, results showed that SR18292 significantly inhibited DOK growth in vivo in the SR18292 group compared with dimethyl sulfoxide (DMSO) groups (Fig. 1j–l). Notably, the administration of SR18292 did not change the body weight (Supplementary Fig. S1b). Histopathological analysis showed high safety of SR18292 in vivo with no damage of tissue (heart, liver, spleen, lung or kidney) in mice treated with DMSO and 45 mg/kg SR18292 (Supplementary Fig. S1c). Therefore, our data demonstrated that SR18292 inhibited DOKs proliferation rates without causing tissue damage.

### Knockdown of PGC1α induces cell cycle arrest of DOKs

**Fig. 2:**
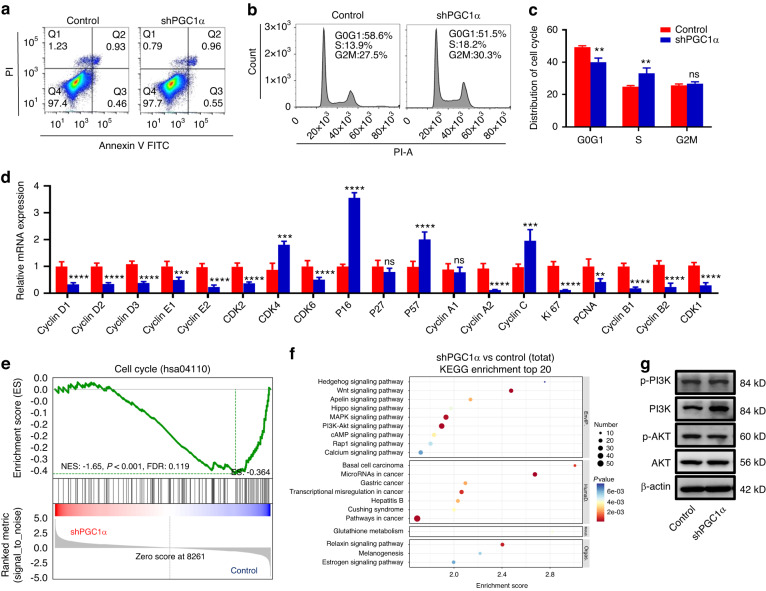
Suppression of PGC1α expression in DOKs-induced cell cycle arrest. **a** Cell apoptosis was detected by flow cytometry. **b**, **c** Cell cycle distribution in control or shPGC1α cells was detected by flow cytometry (**b**) and percentages of cells in the G0G1, S, and G2M phases (**c**). **d** mRNA expression levels of cell cycle-related genes in the control or shPGC1α group. **e** The cell cycle was analyzed using GSEA assays in the control or shPGC1α group. **f** Enrichment plots of the KEGG pathway analysis with the top 20 enrichment score. **g** Western blot analysis was performed for the protein level of PI3K/Akt signaling pathway in the control or shPGC1α group. ns not significant; *P < 0.05, **P < 0.01, ***P < 0.001, ****P < 0.000 1

Suppression of PGC1α expression in DOKs inhibits proliferation. Whether such inhibition effects resulted from cell apoptosis needs further investigation. Our results displayed that suppression of PGC1α did not affect cell apoptosis, as evidenced by no changes in the expression of BAX, Bcl2, and caspase3 (Fig. 2a and Supplementary Fig. S2a). Next, we asked whether this inhibitory effect was related to cell cycle arrest. Flow cytometry assay demonstrated that shPGC1α DOKs displayed S-phase arrest (Fig. 2b, c). To verify the cell cycle arrest, RT-PCR analysis showed that cyclin-dependent kinase (*CDK1*, *CDK2*, *CDK6*) and cyclin (*cyclin A2*, *cyclin B1*, *cyclin B2*, *cyclin D1*, *cyclin D2*, *cyclin D3*, *cyclin E1*, *cyclin E2*) gene expression levels were decreased; in contrast, cyclin-dependent kinase inhibitor (*P16*, *P57*) gene expression levels were upregulated (Fig. 2d and Supplementary Fig. S2b). In addition, gene set enrichment analysis (GSEA) and a heatmap of the analyzed gene sets revealed a considerable reduction of cell cycle and DNA replication gene sets in shPGC1α compared to control DOK; RT-PCR further verified that other cell cycle-related genes were decreased in the shPGC1α group (Fig. 2e and Supplementary Fig. S2c–g). Furthermore, the Kyoto Encyclopedia of genes and genomes (KEGG) analysis was performed to determine the effects of transcriptomic changes on biological functions and pathways. Our findings indicate that the PI3K/Akt signaling pathway was strongly associated with increased proliferation in the shPGC1α group (Fig. 2f). Then we tested the mRNA and protein levels of the PI3K/Akt signaling pathway. Our results showed that PI3K/Akt signaling pathway was reduced in the shPGC1α group (Fig. 2g and Supplementary Fig. S2h). These results demonstrated that the decreased expression of PGC1α induced S-phase arrest was associated with the suppression of the PI3K/Akt signaling pathway.

Next, we explored the effect of SR18292 on the cell cycle distribution, DOKs were exposed to SR18292 (0–75 μmol·L^−1^) for 24 h, which led to the accumulation of S-phase cells (Supplementary Fig. S3a). These phenomena coincided with the decreased S-phase arrest gene levels of cyclin-dependent kinase and cyclins, upregulated the cyclin-dependent kinase inhibitor-related genes expression (Supplementary Fig. S3b, c). These results indicated that SR18292 blocked the S-phase transition of DOKs.

### Knockdown of PGC1α induces OXPHOS dysfunction in DOKs

**Fig. 3:**
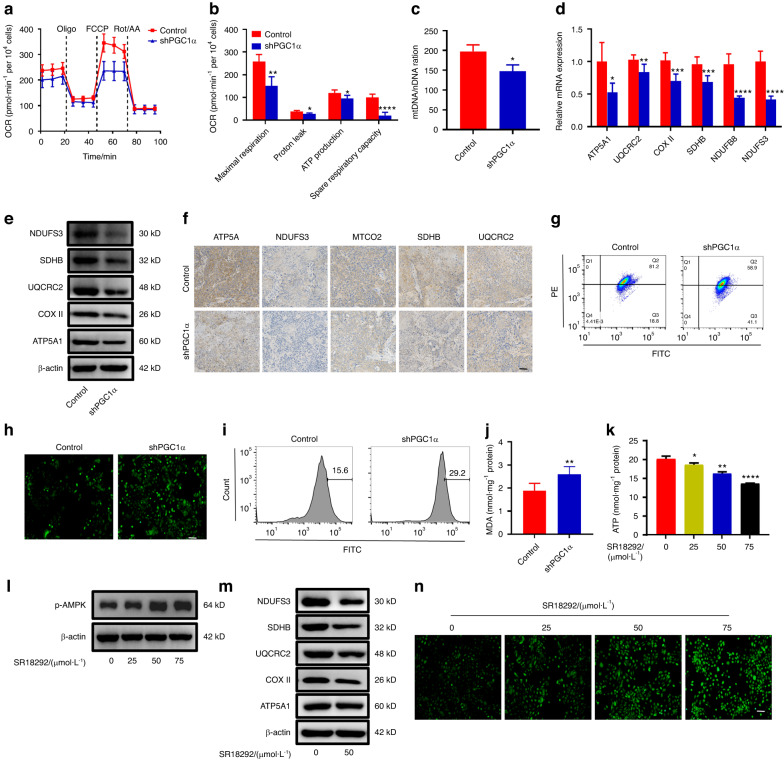
Knockdown PGC1α induces OXPHOS dysfunction in DOKs. **a**, **b** Representative plots (**a**) and quantitative results (**b**) of the cellular OCR measured by Seahorse in the control and shPGC1α DOKs. **c** Relative quantification was performed on control and shPGC1α DOKs using RT-PCR by amplifying of ND1 genes and normalizing against H19 genes. **d**, **e** mRNA (**d**) and protein (**e**) levels of mitochondrial electron transport chain complex subunits in the control and shPGC1α DOKs. **f** Representative immunohistochemistry (IHC) images showing the expression of OXPHOS complex subunits in mice xenografts tumor tissues from control and shPGC1α group, the scale bar indicates 100 μm. **g** Cells were stained with JC-1 and analyzed by flow cytometry. **h**, **i** ROS were analyzed by fluorescence microscope (**h**) and flow cytometry (**i**), the scale bar indicates 100 μm. **j** MDA levels in the control and shPGC1α DOKs. **k** Effect of SR18292 (0–75 μmol·L^−1^) on cellular ATP levels in DOK cells. **l** Western blot analysis of the level of p-AMPK in DOKs treated with SR18292 (0–75 μmol·L^−1^). **m** Western blot analysis of the expression levels of ETC subunits in DOKs treated with DMSO or 50 μmol·L^−1^ SR18292. **n** Represents ROS images were captured by fluorescence microscope in DOKs at 24 h following SR18292 (0–75 μmol·L^−1^) treatment, the scale bar indicates 100 μm. *P < 0.05, **P < 0.01, ***P < 0.001, ****P < 0.000 1

PGC1α has been identified as the primary regulator of mitochondrial biogenesis in various types of cancer, suggesting its critical role in this process. We aimed to determine if downregulated of PGC1α led to reprogramming energy metabolism in DOKs. To test this hypothesis, we performed Seahorse extracellular flux assays. As shown in Fig. 3a, b, XF Cell Mito Stress Test Kit results revealed OCR, maximal respiration, proton leak, and spare respiratory capacity decreased in shPGC1α compared to the control group, indicating that endogenous expression of PGC1α is essential for maintaining mitochondrial respiration in DOKs. Next, we assessed whether there were changes in mitochondrial number and function. RT-PCR results showed that the level of mtDNA decreased in the shPGC1α group compared to the control group (Fig. 3c). Mito-Tracker fluorescence also showed a reduced mitochondrial number in the shPGC1α group (Supplementary Fig. S4a). If this decreased mtDNA truly reflected mRNA and protein levels of the different complexes of the mitochondrial electron transport chain (ETC), our results revealed that NADH: ubiquinone oxidoreductase core subunit S3 (NDUFS3), succinate dehydrogenase complex iron sulfur subunit B (SDHB), ubiquinol-cytochrome c reductase core protein 2 (UQCRC2), cyclooxygenase-2 (COX II), and ATP synthase F1 subunit alpha (ATP5A1) were significantly decreased in the shPGC1α group compared to the control group (Fig. 3d, e). Interestingly, decreased mitochondrial ETC subunits were also observed in vivo (Fig. 3f). Several nuclear transcription factors and coactivators in the regulation of mitochondrial biogenesis were reduced, such as mitochondrial transcription factor A (TFAM), nuclear respiratory factor 1/2 (NRF1/NRF2), and the PGC family of transcriptional coactivators (*PGC1β*, *PRC*) were indeed downregulated (Supplementary Fig. S4b, c). These results suggested that suppression of PGC1α expression in DOKs damaged mitochondrial biogenesis.

Mitochondrial membrane potential (MMP) drives mitochondrial ATP generation via coupling oxygen consumption with ATP production. Flow cytometry analysis and fluorescence demonstrated decreased MMP in shPGC1α compared to control DOKs, indicating lower ATP production (Fig. 3g and Supplementary Fig. S4d). AMPK as a sensor of cellular energy, which is activated by phosphorylation when the cellular ATP content decreases,^28^ our results showed that the p-AMPK increased in the shPGC1α group (Supplementary Fig. S4c). Previous studies have reported that reactive oxygen species would be overproduced if the OXPHOS complexes were defective. Furthermore, ROS was higher in shPGC1α with decreased antioxidant genes (superoxide dismutase 1 (*SOD1*), catalase (*CAT*), and glutathione peroxidase 1/3 (*GPX1/3*)), and the high MDA level was consistent with the ROS level (Fig. 3h–j and Supplementary Fig. S4e).

To further evaluate the effect of SR18292 on ATP production. We found that the MMP declined in DOKs by fluorescence when the concentration of SR18292 increased from 0 to 50 μmol L^−1^ (Supplementary Fig. S4f). And SR18292 induced a marked decrease in ATP production in DOKs (Fig. 3k), but p-AMPK was activated (Fig. 3l). Next, we measured the effects of SR18292 on the number of mitochondrial and the expression of OXPHOS genes. Mito-Tracker fluorescence showed mitochondrial number decreased in SR18292 (50 μmol·L^−1^) compared with the DMSO group (Supplementary Fig. S4g). And SR18292 significantly reduced in the expression of OXPHOS subunits genes and proteins in vitro and in vivo (Fig. 3m and Supplementary Fig. S4h, i). Finally, we measured the ROS levels after treatment with SR18292 (0–75 μmol·L^−1^), and results showed that the intracellular ROS level was markedly increased in a concentration-dependent manner in DOKs (Fig. 3n and Supplementary Fig. S4j). Together, these results indicated that SR18292 induced an energy crisis in DOKs.

### Knockdown of PGC1α enhances glycolytic phenotype in DOKs

**Fig. 4:**
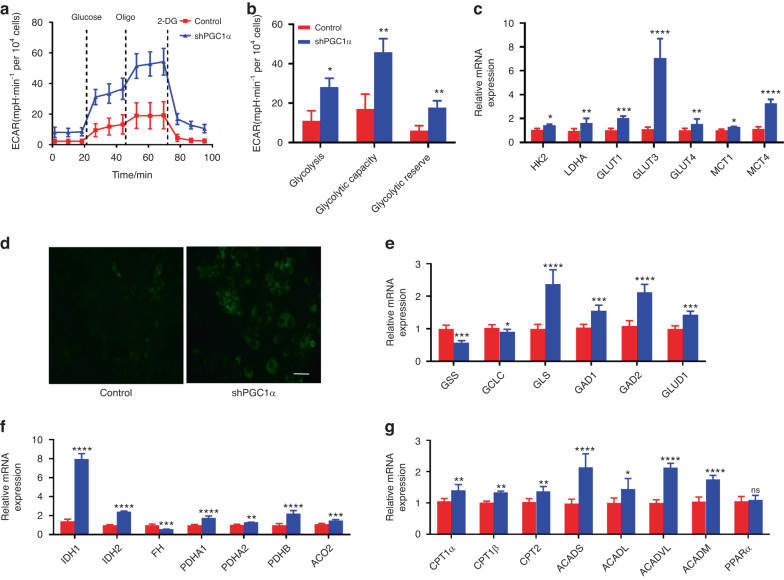
Knockdown PGC1α enhanced the glycolytic phenotype of DOKs. **a**, **b** Representative plots (**a**) and quantitative results (**b**) of the real-time ECAR assays by Seahorse in the control and shPGC1α DOKs. **c** mRNA levels of glycolytic, glucose transporter, and monocarboxylate transporter genes in the control and shPGC1α DOKs. **d** Glucose uptake was assayed with 2-NBDG by fluorescence microscope, the scale bar indicates 50 μm. **e**–**g** mRNA levels of glutamine (**e**), tricarboxylic acid cycle (**f**) and fatty acid metabolism (**g**) genes in the control and shPGC1α DOKs. ns not significant; *P < 0.05, **P < 0.01, ***P < 0.001, ****P <0.000 1

Then we asked whether there were other metabolic changes to provide energy. This question prompted us first to investigate glycolysis. XF Cell Glycolysis Stress Test Kit results showed ECAR, glycolysis, glycolytic capacity, and glycolytic reserve were significantly higher in shPGC1α than in the control group (Fig. 4a, b). We next assessed whether the expression of glycolysis enzymes (hexokinase, pyruvate kinase, and LDH) was increased in shPGC1α group. As shown in Fig. 4c, *HK2* and *LDHA* expressions were significantly increased. Moreover, we also found increased expression of glucose transporter 1/3/4 (*GLUT1/3/4*) and lactate exporter monocarboxylate transporter 1/4 (*MCT1/4*, Fig. 4c). The increased *GLUT1/3/4* expression implies an increase in glucose uptake. To test this, we incubated the shPGC1α and control group DOKs with the fluorescent 2-NBDG. As shown in Fig. 4d, the fluorescence intensity is significantly increased in shPGC1α DOKs, indicating an increase in glucose uptake. These results demonstrate that PGC1α knockdown enhanced DOKs’ glycolysis to provide energy.

Besides glycolysis, we investigated the expression of several enzymes involved in the tricarboxylic acid cycle (TCA), glutamine, and fatty acid metabolism using RT-PCR. Glutamine produces glutamate through the deamidation of glutaminase (GLS). Endogenous glutamate can either feed the TCA cycle after conversion into α-KG by glutamate dehydrogenase 1 (GLUD1) or converted into GABA by the action of glutamic acid decarboxylase (GAD). As shown in Fig. 4e, the mRNA levels of *GLS*, *GAD1/2*, and *GLUD1* were significantly increased, indicating that glutamine metabolism was enhanced. Although the pyruvate dehydrogenase E1 subunit alpha 1/2 (*PDHA1/2*), pyruvate dehydrogenase E1 subunit beta (*PDHB*), aconitase 2 (*ACO2*), isocitrate dehydrogenase (NADP( + )) 1/2 (*IDH1/2*) were also significantly increased, the mitochondrial bioenergetics was low due to the decreased several subunits of ETC (Fig. 4f). For lipid metabolism, we observed carnitine palmitoyltransferase 1α/β (*CPT1α/β*), *CPT2*, acyl-CoA dehydrogenase short-chain (*ACADS*), acyl-CoA dehydrogenase long chain (*ACADL*), acyl-CoA dehydrogenase very long chain (*ACADVL*), acyl-CoA dehydrogenase medium chain (*ACADM*) were significantly increased in shPGC1α DOKs (Fig. 4g). These results indicated that other metabolic pathways were involved in energy metabolism when OXPHOS was impaired.

### Knockdown PGC1α enhances the glycolytic with upregulating LDHA in DOKs

**Fig. 5:**
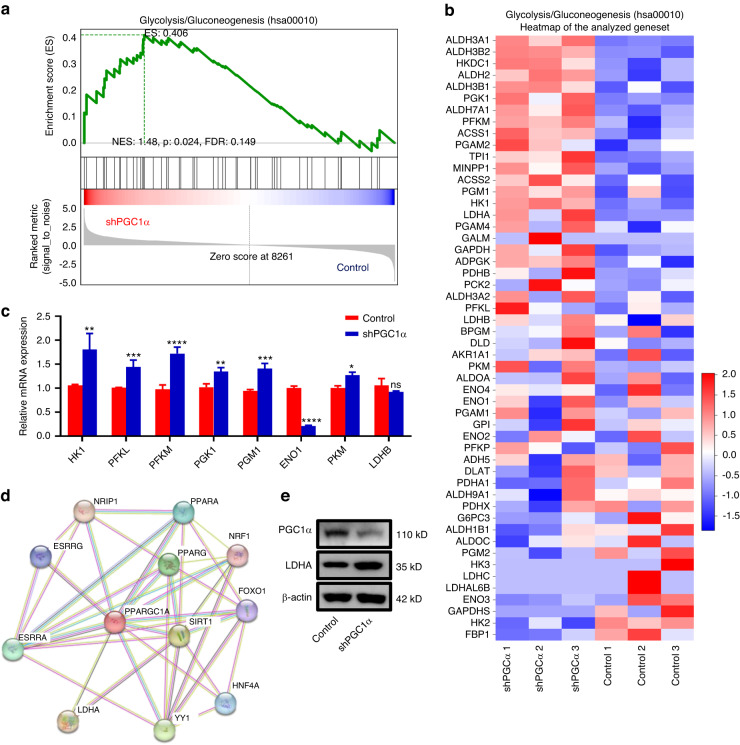
PGC1α knockdown enhances the glycolytic in DOKs by upregulating LDHA. **a** Glycolysis/gluconeogenesis was analyzed using GSEA assays in the shPGC1α or control group. **b** Heatmap of the analyzed geneset of glycolysis/gluconeogenesis gene sets in the shPGC1α or control group. **c** RT-PCR analysis of the cell cycle gene in (**b**). **d** The association networks between PGC1α and LDHA gene were got form the Serach Tool for the Retrieval of Interacting Genes (STRING) database. **e** Western blot analysis was performed for the protein level of PGC1α and LDHA in DOKs in the shPGC1α or control group. **P < 0.01, ***P < 0.001, ****P < 0.000 1

Several reports have shown that LDH catalyzes the interconversion between pyruvate and lactate.^29–31^ PGC1α can synergistically change the composition of the LDH complex and control metabolic adaptations. PGC1α in skeletal muscle drives the expression of LDHB and reduces the expression of LDHA.^32^ Therefore, to better characterize the molecular pathways involved in the metabolic changes, we asked whether PGC1α and LDHA play a role in the metabolic changes in DOKs. Consistent with impaired mitochondrial function in shPGC1α DOKs, GSEA analysis revealed glycolysis/gluconeogenesis enrichment score increases in shPGC1α (Fig. 5a). Heatmap analysis and RT-PCR also showed increased glycolysis/gluconeogenesis related genes (*HK1*, *PFKL*, *PFKM*, *PGK1*, *PGM1*, *PKM*, *LDHA*) (Fig. 5b, c). Interestingly, we found an association network between PGC1α and LDHA, further confirming the negative correlation between PGC1α and LDHA (Fig. 5d). Western blot results also confirmed this result (Fig. 5e). Taken together, these results indicated that knockdown PGC1α enhances the glycolytic with upregulating LDHA in DOKs.

## Discussion

Energy and biosynthetic precursors are necessary for supporting anabolic reactions in the rapid proliferation of cancer cells. In our study, we found that knockdown PGC1α significantly inhibited the proliferation of DOKs in vitro and xenograft tumor growth in vivo. It is a well-established function of PGC1α in regulating of mitochondrial energy metabolism in cancer cells;^33^ the knockdown of PGC1α in our study significantly reduced the mitochondrial mass, mitochondrial respiration complex proteins, and OCR, but increased glycolysis and other metabolism pathways. Our results suggest that changes in mitochondrial function occur in the precancerous state and may continue to worsen, thus contributing to the enhancement of various malignant biological capabilities of cancer cells. In accordance with shRNA-mediated knockdown, PGC1α had impaired proliferation and migration rates in glioblastoma cells.^34^ And in melanoma cells, a low level of PGC1α is more glycolytic, decreasing the proliferation and survival against ROS-induced apoptosis.^35^

Our results showed that the PI3K/Akt signaling pathway was decreased in shPGC1α group DOKs, suggesting that PI3K/Akt may be a downstream effector of PGC1α. PI3K/Akt signaling pathway has diverse effects on cell proliferation, growth, differentiation, and motility.^36^ Genes of this pathway were found to be commonly activated and contributed to the occurrence and progression of tumors.^37,38^ Knockdown of PGC1α abrogated hypoxia-induced pulmonary arterial vascular smooth muscle cells proliferation via the downregulation of PCNA, cyclinA, and cyclinE and involved in PI3K/Akt signaling.^39^ In androgen-dependent prostate cancer cells, knockdown PGC1α induces G1-phase arrest and thus reduces their growth in vitro.^40^ PGC1α also acts as the co‑activators of ERRα and activates ERRα via promoting PI3K/Akt phosphorylation to promote the proliferation and invasion of gallbladder cancer cells.^41^ Another study in human colorectal cancer revealed that knockdown PGC1α inhibited cell proliferation via the AKT/GSK-3β/β-catenin pathway.^42^

In addition, PI3K/Akt signaling pathway affects cell energy metabolism by either regulating metabolic enzymes or metabolic pathways.^43^ PI3K signaling pathway enhances glycolytic flux in an Akt-independent/dependent manner. Akt also promotes glucose uptake through both GLUT1 and GLUT4, then activation of specific glycolytic enzymes in glycolysis.^43,44^ In thyroid cancer, PGC1α knockdown was inversely related to AKT activity, induced a glycolytic phenotype and suppressed tumor growth.^17^ However, the expression of PI3K and Akt mRNA/protein level is decreased in shPGC1α cell in our study, and the glycolysis enzymes (HK1/2, PKM, LDHA), GLUT1/3/4, glucose uptake is higher, which indicted that PI3K/Akt signaling pathway not involved in regulating glycolysis.

LDHA is one of the key metabolic enzymes that facilitates the rapid glycolytic process by converting pyruvate to lactate. Aberrant expression and activation of LDHA were closely related to diverse diseases.^45^ PGC1α can remodel LDH isoenzyme composition in skeletal muscle, which reduces the expression of LDHA and increase the expression of LDHB.^32^ Shu et al. study also demonstrated that transforming growth factor-beta 2 (TGFβ2) suppressed PGC1α expression in retinal pigment epithelial cells, induced defects in mitochondrial network integrity, and increased the glycolytic enzymes (PKM2, LDHA) expression.^46^ Our results showed that the expression of LDHA is higher in shPGC1α DOKs. LDHA expression levels in tumor tissues of head and neck squamous cell carcinoma (HNSCC) patients were significantly higher than healthy tissue, and associated with a poor disease-free survival. LDHA expression induces epithelial-mesenchymal transition (EMT) and promotes cell proliferation, invasion, and migration in HNSCC cell lines.^47–49^ The expression of LDHA was significantly increased in OSCC comparison to normal tissue and hyperplasia; no difference is observed between OSCC and squamous intraepithelial neoplasia I-III.^50^ Besides, knockdown LDHA inhibited esophageal squamous cell carcinoma cell growth and cell migration in vitro, decreased the expression of cyclin D1, and increased cleavage of PARP and caspase 8 expressions.^51^ Several other reports highlighted that LDHA contributes to cancer metastasis by activating EMT-related genes, accompanied by decreased E-cadherin expression, and increased Snail, N-cadherin, fibronectin, and vimentin expression.^52,53^ Therefore, LDHA helps cancer cells to establish and proliferate by enhancing angiogenesis, increasing cell motility, migration and invasion, promoting EMT, and favoring the tumor immune escape, which will help us explore cancer pathogenesis and its handling measures.^30^

Endogenous ROS mainly occurs in mitochondrial respiratory chain complex I and III, and is necessary for normal physiology and the development of various diseases, including cancer.^54–57^ PGC1α can potently increase antioxidant enzymes such as manganese superoxide dismutase (MnSOD), CAT, and peroxiredoxin 3/5, consequently reducing ROS production and protecting of cells from mitochondrial dysfunction.^58–60^ At the same time, ROS production can also trigger PGC1α expression.^61^ Generally, our results have indicated that the downregulation of PGC1α in DOKs significantly inhibits the expression of OXPHOS genes and decreases the expression of several antioxidant genes such as CAT, GPX1/3, and SOD1, resulting in rapid ROS production, but without inducing apoptosis.

ROS is a “double-edged sword” in cancer cells.^62^ In our studies, ROS is higher in the shPGC1α group and SR18292 group. At the precancerous/early stage of tumor progression, low to moderate ROS levels initiates extensive oxidative damage and adaptation reaction in the cell components, which leads to DNA damage and mutations in pro-oncogenes or tumor suppressor genes, subsequently activating the cancer cell survival signaling and generate an inflammatory environment, thereby augment tumorigenesis, development, progression, metastasis, and survival.^63,64^ In advanced stages of cancer progression, tumor cells produce high levels of antioxidants to buffer the overproduction of ROS, reduce intra-tumor oxidative injuries and escape apoptosis.^65,66^ In contrast, other studies have shown that the overproduction of ROS leads to the damage of proteins, nucleic acids, lipids, and organelles, which in turn activates the cell death signaling pathways, causing cell cycle arrest, senescence, and apoptosis.^55,67^ This increases the understanding of the complexity of ROS in carcinogenesis and enables the uncovering of the potential of ROS-targeting therapies for cancer.

PGC1α activation and expression are influenced by modulation of its expression level, its binding partners’ availability and functional status, and posttranslational modifications.^14,68^ SR18292, a selective pharmacological inhibitor, induces PGC1α acetylation and suppresses PGC1α-dependent gluconeogenic gene expression in mice.^69^ Thus, we analyzed the effects of treatment with SR18292 on DOKs. Remarkably, the impact of SR18292 markedly reduced total ATP, the number of mitochondrial, mRNA/proteins expression of OXPHOS subunits and the volume of tumor xenografts, and increased AMPK phosphorylation and ROS production. Consistent with Xiang et al. results, SR18292 led to oxidative damage and energy exhaustion, significantly inhibited the proliferation of multiple myeloma cells, and inhibited tumor growth in myeloma model mice.^70^ Similarly, in cholangiocarcinoma, SR18292 also reduced PGC1α levels, mitochondrial mass, and mice with tumor xenografts growth.^71^ Collectively, these data indicated that SR18292 induced an energy crisis, which can be employed as a pharmacological target for cancer treatment.

OPMDs refer to any oral mucosal abnormality that increases the risk of developing oral cancer. This group of lesions has varying rates of malignant transformation depending on ethnic, genetic, geographic, and lifestyle factors.^25,72^ Cancer cells are able to reprogram their metabolism, and PGC1α plays crucial roles in regulating adaptive thermogenesis, mitochondrial biogenesis, oxidative phosphorylation, muscle fiber-type switching, and gluconeogenesis. It affects tumor development by regulating various metabolic pathways. This study explored the mechanisms of PGC1α in DOKs’ biological behavior and energy metabolism. The results displayed that knockdown PGC1α in DOKs significantly inhibited the proliferation and tumor growth, induced S-phase arrest with suppressed PI3K/Akt signaling pathway. Mechanistically, downregulated of PGC1α decreased OCR and the expression of mitochondrial electron transport chain complexes and enhanced glycolysis by upregulating LDHA, glucose uptake, and ECAR. In addition, SR18292 also induced DOKs OXPHOS dysfunction and slowed DOK xenograft progression. In-depth molecular studies of PGC1α in OPMDs may lead to the development of targeted drugs. Still it is crucial to consider the role of PGC1α in normal physiological functions of the body and to conduct a comprehensive evaluation during targeted therapy. However, it remains to be determined whether the effect of PGC1α on LDHA expression is a direct transcriptional effect or secondary to the induction of glycolysis. Future studies will elucidate the mechanisms between PGC1α and LDHA in OPMDs regulating energy metabolism and distinct or related transcriptional programs. In addition, we will also need to focus on the relationship between PGC1α and other metabolism pathways in OPMDs.

## Materials and methods

### Chemicals or reagents

The extracellular acidification rate (ECAR) and oxygen consumption rate (OCR) assay kits were purchased from Agilent (Seahorse Biosciences, North Billerica, USA). Other reagents are listed in Supplementary Table S1.

### Cell culture and establishment of PGC1α knockdown DOKs

DOKs were provided by the laboratory and tested to confirm no mycoplasma infection. DOKs were cultured in DMEM (Hyclone, Logan, USA), supplemented with 100 U·mL^−1^ penicillin, 100 g·L^−1^ streptomycin (Hyclone, Pasching, USA), 5 μg/ml hydrocortisone, and 10% fetal bovine serum (FBS, Gibco, USA). Cells were cultured in a humidified atmosphere of 95% air/5% CO_2_ at 37 °C. shPGC1α lentivirus and control lentivirus were purchased from GeneChem (Shanghai, China). DOKs infected with the lentiviral stocks according to the manufacturer’s protocol. Puromycin (5 g·L^−1^) was added to the culture medium to select successfully transfected cells. PGC1α knockdown DOKs are termed shPGC1α cells, and control cells generated with a nontargeting shRNA sequence are termed control in this paper.

### Real-time PCR (RT-PCR)

Total RNA was isolated from cell lines using the Trizol reagent according to the manufacturer’s instructions. In general, 1 μg total RNAs was reverse transcribed into cDNA with PrimeScript™ RT reagent Kit with gDNA Eraser. Diluted cDNAs were analyzed for qPCR using SYBR Green PCR Master Mix (Applied Biosystems) and gene-specific primers (Supplementary Table S2). β-actin gene expression was used as an endogenous control. The relative expression ratio is presented as fold change according to the 2^-ΔCt^ method.

### Mitochondrial DNA quantification

Total DNA was isolated utilizing a Genomic DNA kit. The level of mitochondrial DNA genome (mt-ND1) and nuclear gene (H19) was amplified with primers provided in Supplementary Table S2.

### RNA sequencing analysis

Total RNA extracted from DOKs with or without PGC1α knockdown was subjected to RNA sequencing (Oebiotech, Illumina NoVaSeq6000). For the Kyoto Encyclopedia of Genes and Genomes (KEGG) analysis, differentially expressed mRNA with log2 (Fold change) >1 were included, and *P* < 0.05 was considered as significant difference. The sequencing data was uploaded to the GEO database (GSE224317).

### Western blot

Cells were lysed in RIPA lysis and extraction buffer (Solarbio) with protease and phosphatase inhibitor mixture (Beyotime, China). Protein concentrations were measured by the bicinchoninic acid assay. An equal amount of protein extracts were resolved on SDS-polyacrylamide gel and electro-blotted onto polyvinylidene difluoride (PVDF) membranes (Bio-Rad, USA). The primary/ appropriate secondary antibodies were listed in Supplementary Table S3. All antibodies were diluted in 5% skim milk or bovine serum albumin (BSA) in TBST. Nonspecific binding sites were blocked with 5% skim milk or BSA for 1 h at room temperature. Next, the samples were incubated overnight at 4 °C with the primary antibody. On day two, membranes were incubated with the appropriate secondary antibodies for 1 h at room temperature. Finally, the immunocomplexes were visualized by enhanced chemiluminescent substrates (Millipore, USA), and the intensity of the chemiluminescence response was analyzed by ImageJ.

### Cell counting kit-8 (CCK-8) assay

In total, 1 × 10^3^ cells were seeded in 96 well plates at 0.2 mL suspension per well, incubated with or without various compounds at 37 °C. Then, after culture for 24, 48, 72, 96, and 120 h, 10 μL CCK-8 solution was added to each well and incubated at 37 °C for 1 h, and the absorbance at 450 nm was recorded.

### Colony-formation assay

Cells were seeded in six‐well plates at 2 × 10^3^ cells per well. After 14 days of culture, cells were fixed with 4% paraformaldehyde, and stained with 0.1% crystal violet. Colonies were captured by light microscope and counted using ImageJ.

### Cell cycle

The cell cycle was evaluated by DNA content quantitation assay kit according to the manufacturer’s protocol. Cells were collected, washed and fixed with 70% ethanol at −20 °C overnight, and then add 100 μL RNase A for 30 min at 37 °C water bath, stained with 400 μL PI dye for 30 min in the dark at 4 °C. Finally, cells were analyzed by flow cytometry and data were analyzed by FlowJo software (Becton, Dickinson and Company, USA).

### Apoptosis was detected by flow cytometry

Annexin-V-FITC apoptosis detection kit was used to detect apoptosis following the manufacturer’s protocol. Cells were collected, washed and then resuspended in 500 μL binding buffer, stained with 5 μL of Annexin-V-FITC and 5 μL of PI for 15 min, followed by flow cytometry analysis. FlowJo was used to analyze the flow cytometry data.

### Detection of ROS

ROS was measured by staining the cells with the cell-permeable DCFH-DA. Cells were washed with PBS and incubated with 10 μmol·L^−1^ DCFH-DA dissolved in DMEM without serum. After incubation for 20 minutes, cells were washed with DMEM without serum. The fluorescence signal was measured by Olympus fluorescence FV3000 microscope or flow cytometry.

### Determination of malondialdehyde (MDA) levels

The concentration of MDA was measured using the lipid peroxidation MDA assay kit following the manufacturer’s protocol. Briefly, 100 μL of the cell lysates was mixed with 200 μL MDA working solution, at 100 °C for 15 min, and then cooled down to room temperature. After centrifugation at 1 000× *g* for 10 min, the supernatant was measured at a wavelength of 532 nm. MDA levels were calculated according to the established standard curve (unit was expressed as nmol/mg of protein).

### MMP measurement

MMP was measured with a mitochondrial membrane potential assay kit. Cells were seeded in six-well plates, after a period of culture, then incubated in fresh medium containing JC-1 for 20 min at 37 °C. Finally, cells were washed twice and then subjected to flow cytometry analyses.

### Cellular ATP measurement

ATP test using an ATP assay kit according to the manufacturer’s instructions. 100 μL ATP assay buffer was added to each well, and incubated for 3 min before adding 20 μL cell lysates to each well, mixed, and luminescence was measured using a luminometer (Molecular Devices). ATP levels were calculated according to the established standard curve and were generated using a series of known concentrations of ATP standard solutions (1–50 nmol·L^−1^).

### Glucose uptake assay

Glucose uptake was monitored using fluorescent 2-NBDG. Cells were washed with PBS, incubated for 2 h at 37 °C in DMEM (5 mmol·L^−1^ glucose) without serum, and then coincubated with 100 μmol·L^−1^ 2-NBDG for 30 min in the dark at 37 °C. The supernatant was removed, and cells were washed twice with PBS. Fluorescent images were taken with an Olympus fluorescence FV3000 microscope.

### OCR and ECAR determination

OCR/ECAR was measured with a Seahorse XFe 24 Extracellular Flux Analyzer according to the manufacturer’s instructions. Briefly, the control (2 × 10^4^ cells) and shPGC1α (2 × 10^4^ cells) were seeded per well in an XFe 24 plate with complete DMEM medium for 24 h. Before 1-h detection, the medium was changed to Seahorse XF Base Medium. OCR was tested using the compounds of oligomycin (1.5 μmol·L^−1^), FCCP (1 μmol·L^−1^), and rotenone and antimycin A (each 0.5 μmol·L^−1^). ECAR was monitored using the compounds of glucose (100 mmol·L^−1^), oligomycin (10 μmol·L^−1^), and 2-deoxyglucose (500 mmol·L^−1^). The results were normalized to the number of cells in each plate determined at the time of measurement.

### Subcutaneous xenograft models

Animal experiment was approved by the ethics committee of the West China Hospital, Sichuan University (20220922002). Female BALB/C nude mice (aged 4–6 weeks) were purchased from Beijing HFK Bio-Technologies. Animals were randomly divided into control and shPGC1α groups (*n* = 4 per group) and then subcutaneously injected with 0.2 mL of cell suspension containing 5 × 10^6^ cells. After 2 weeks when a tumor was palpable, control and shPGC1α group mice were measured twice a week, and its volume was calculated using the formula volume = 0.5 × length × width^2^. All mice were sacrificed 4 weeks later; then tumors were excised, weighed, and fixed in 4% paraformaldehyde for further analysis.

In total, 5 × 10^6^ DOKs were subcutaneously injected into mice. When the tumor volume reached ~100 mm^3^, the mice were randomly divided into the DMSO group (5% DMSO) and SR18292 group (45 mg·kg^−1^ in 5% DMSO, *n* = 5 per group) and received daily intraperitoneal injection for 15 days, respectively. The size of the xenograft tumor and body weight were measured every day. Tumors were excised and weighed on day 15, and mice’s hearts, livers, lungs, spleens, and kidneys were fixed in 4% paraformaldehyde.^70^

### Immunohistochemistry evaluation

All tissues fixed in 4% paraformaldehyde solution were embedded in paraffin and sectioned (4-μm thick) with a rotary microtome. Immunostaining for PGC1α, MTCO2, NDUFS3, UQCRC2, ATP5A1, and SDHB was performed according to standard procedures. Sections were viewed with a light microscope.

### Statistics

In vitro experiments were done in triplicates if not otherwise noted, and one representative experiment is shown. Results are presented as means ± standard deviation (SD), and GraphPad Prism Software 8.0 was used for statistical analyses and graphs. Comparisons between the two groups were performed with the unpaired Student’s *t* test. Comparisons among multiple groups were conducted with one-way analysis of variance. And *P* <0.05 was considered to be statistically significant.

### Supplementary information

Supplementary information

Fig. S1

Fig. S2

Fig. S3

Fig. S4

## Data Availability

The data presented in this study are available upon request from the corresponding author.
