# Supplementary material for: Knockdown of PGC1α suppresses dysplastic oral keratinocytes proliferation through reprogramming energy metabolism
Source: Int J Oral Sci. 2023 Sep 4;15:37. doi: 10.1038/s41368-023-00242-3 (PMC10475463; doi:10.1038/s41368-023-00242-3)
Supplement: Supplementary file 1 — Supplementary information [file 41368_2023_242_MOESM1_ESM.docx]

**Title:** **Knockdown of PGC-1α suppresses dysplastic oral keratinocytes proliferation through reprogramming energy metabolism**

Authors: Yunkun Liu, Nengwen Huang, Xianghe Qiao, Zhiyu Gu, Yongzhi Wu, Jinjin Li, Chengzhou Wu, Bo Li,*, Longjiang Li,*

**Supplemental Appendix**

**MATERIALS AND METHODS**

Histology evaluation

All tissues fixed in 4% paraformaldehyde solution were embedded in paraffin. Embedded samples were sectioned (4 μm thick) with a rotary microtome and stained with hematoxylin and eosin (H&E) according to established protocols. Sections were viewed with a light microscope.

Mito-tracker

Cells were seeded in confocal dish, after 24h, incubated with a staining solution made of mito-tracker red CMXRos (200 nmol•L^-1^) in medium for 30 minutes, then fixed with 4% paraformaldehyde for 20 min, add mounting medium, antifading (with DAPI). Cell images were captured using a FV3000 confocal laser-scanning fluorescence microscope (Olympus, Japan).

Mitochondrial membrane potential (MMP) measurement

MMP was measured with mitochondrial membrane potential assay kit. Cells were seeded in 6-well plates, with or without treatment, cells were cultured in fresh medium containing JC-1 for 20 min at 37 ℃. Finally, cells were washed twice and then subjected to the analyses of Olympus fluorescence microscope.

**Fig. S1** Suppression of PGC1α expression in DOKs without affecting the weight of mice. **a** Weight of xenograft tumors mice in control and shPGC1α DOKs (n = 4). **b** A xenograft tumors from DOKs tumor-bearing mice injected with 5% DMSO or SR18292 (45 mg/kg, n = 5) daily for 15 days. **c** H&E staining analysis of organ tissue damage in mice treated with DMSO and SR18292, the scale bar indicates 100 μm.

**Fig. S2** Effects of PGC1α knockdown on proliferation, apoptosis and cell cycle of DOKs. **a** Western blot analysis was performed for Bax, Bcl2, Caspase3 in control or shPGC1α group. **b** Western blot analysis was performed for PCNA and P16 in control or shPGC1α group. **c-e** GSEA assays of DNA replication (**c**), DNA replication initiation (**d**) and DNA replication origin binding (**e**) in control or shPGC1α group. **f** A heatmap of the analyzed gene sets of cell cycle gene sets in control or shPGC1α group. **g** RT-PCR analysis the cell cycle genes in (**f).** **h** mRNA levels of genes involved in PI3K/Akt signaling pathway. **P* < 0.05, ***P* < 0.01, ****P* < 0.001, *****P* < 0.0001.

**Fig. S3** The effect of SR18292 on DOKs cell cycle distribution. **a** The cell cycle distribution was measured by flow cytometry after DOKs were incubated with SR18292 (0-75 μmol•L^-1^) for 24 h. **b** Western blot analysis of P16 in DOKs following exposure to SR18292 (0-75 μmol•L^-1^) for 48 h. **c** RT-PCR analysis of the expression levels of cell cycle-related genes in DOKs treated with DMSO or 50 μmol•L^-1^ SR18292. ns, not significant; **P* < 0.05, ***P* < 0.01, ****P* < 0.001, *****P* < 0.0001.

**Fig. S4** Effects of PGC1α knockdown on the mitochondrial membrane potential and ROS. **a** Mito-tracker analysis the number of mitochondrial in control and shPGC1α DOKs by confocal microscopy, the scale bar indicates 25 μm. **b** mRNA levels of PGC1α, PGC1β, PRC, TFAM, NRF1 and NRF2 genes in control and shPGC1α DOKs. **c** Western blot analysis was performed to detect the protein levels of p-AMPK, AMPK, TFAM, NRF1, NRF2 in control and shPGC1α DOKs. **d** Cells were stained with JC-1 and analyzed by fluorescence microscope, the scale bar indicates 50 μm. **e** mRNA levels of antioxidant genes. **f** Represents MMP images were captured by fluorescence microscope in DOKs after the addition of gradient concentrations of SR18292 for 48 h, the scale bar indicates 50 μm. **g** Mito-tracker analysis the number of mitochondrial in DOK cells treated with DMSO or 50 μmol•L^-1^ SR18292, the scale bar indicates 25 μm. **h** mRNA analysis of the expression levels of ETC subunits in DOKs treated with DMSO or 50 μmol•L^-1^ SR18292. **i** IHC analysis of the expression of ETC subunits in tumor tissues from control or SR18292-treated mice xenografts, the scale bar indicates 100 μm. **j** ROS levels were assessed by flow cytometry in DOKs following a range of concentrations of SR18292 treatment at 24h. **P* < 0.05, ***P* < 0.01, ****P* < 0.001, *****P* < 0.0001.

**Table S1.** Chemicals or Reagents

| Chemicals or Reagents | Sources | Identifier |
| --- | --- | --- |
| 2-NBDG | APExBIO | B6035 |
| Trizol reagent | Life Technologies | 15596026 |
| 0.1% crystal violet | Solarbio | G1063 |
| SR18292 | Selleck | S8528 |
| DMSO | Sigma | D2650 |
| ATP Assay Kit | Beyotime | S0026B |
| Lipid peroxidation MDA assay kit | Beyotime | S0131S |
| Bicinchoninic acid assay | Beyotime | ST2222 |
| Puromycin dihydrochloride | Beyotime | ST551 |
| Hydrocortisone | Solarbio | G8450 |
| Mounting medium, antifading (with DAPI) | Solarbio | S2110 |
| Mitochondrial membrane potential assay kit with JC-1 | Solarbio | M8650 |
| DNA Content Quantitation Assay (Cell Cycle) | Solarbio | CA1510 |
| PrimeScript™ RT reagent Kit with gDNA Eraser | Takara | RR047A |
| Cell counting kit-8 | Biomake | B34304 |
| Genomic DNA kit | Tiangen | DP304 |
| Reactive oxygen species assay kit | Solarbio | CA1410 |
| Annexin V-FITC/PI Apoptosis Kit | KeyGEN | KGA107 |

**Table S2.** Primer sequence of gene

| Gene | Forward primer (5’-3’) | Reverse primer (5’-3’) |
| --- | --- | --- |
| MCM2 | FORWARD | CAGCGATGAGGAGGACGAGGAG |
|  | REVERSE | AAGTTCTTGAAGCGGTGGTGGATC |
| MCM3 | FORWARD | GCCAAGCAGTATGAGGAGTTCTACG |
|  | REVERSE | GTGACAATGCCCTCCACACAGAC |
| MCM4 | FORWARD | TCAGAGACGTAGAGGCGAGGATTC |
|  | REVERSE | GAGTGCCGTATGTCAGTGGTGAAC |
| MCM5 | FORWARD | CAAGGATGAGCACAATGAGGAGAGG |
|  | REVERSE | CACACTTCACTCGGCAGTAGGC |
| MCM6 | FORWARD | CCTGTGAACGGGATCAATGGCTAC |
|  | REVERSE | GCTCGCTCCTCTTTAATGCTGACTC |
| MCM7 | FORWARD | TGGAGATGAGGCGAGAGGCTTG |
|  | REVERSE | CACCACATCCACCATTCTCAGACG |
| CDC6 | FORWARD | AGGCACAGGCTACAATCAGTTTTCC |
|  | REVERSE | CGAGGAGAACAGGTTACGGTTTGG |
| CDC7 | FORWARD | GCCTGACCTGTGACTGCTATGC |
|  | REVERSE | TGAATCCTGGTGTACCTGCCCTAG |
| RB1 | FORWARD | AGGACCGAGAAGGACCAACTGATC |
|  | REVERSE | CTGGAAGGCTGAGGTTGCTTGTG |
| RBL1 | FORWARD | CGTTCCTTGATGGCTTGTTGTTTGG |
|  | REVERSE | GAGCGGATCACCACCTCAATAACC |
| SKP2 | FORWARD | GAGATTCCAGACCTGAGTAGCAACG |
|  | REVERSE | TCTCACTGTCGGGCTCCTCTTTC |
| P18 | FORWARD | ATCCCGATTTGAAAGACCGAACTGG |
|  | REVERSE | CCGTGTGCTTCACCAGGAACTC |
| E2F1 | FORWARD | CTGTGCCCTGAGGAGACCGTAG |
|  | REVERSE | GATGATGGTGGTGGTGACACTATGG |
| E2F2 | FORWARD | CCAAGGGGAAGTGCATCAGAGTG |
|  | REVERSE | CCAGCGAAGTGTCATACCGAGTC |
| E2F3 | FORWARD | CTCTGGAGCAGTACCTGGTGACC |
|  | REVERSE | GTGGTGGAAGTGTTCGTGGTGAG |
| E2F4 | FORWARD | GAGCAAGAACTAGACCAGCACAAGG |
|  | REVERSE | CCAAGAGGGTATCTCCAGCAAAGC |
| DP1 | FORWARD | AGCCTTCCGACTCCTCACCTTG |
|  | REVERSE | GCTCGTCTGCCACTTCGTTGTAG |
| PIK3CB | FORWARD | CTGCCTGCGACAGATGAGTGATG |
|  | REVERSE | ACTGCCCTATCCTCCGATTACCAAG |
| PIK3CD | FORWARD | GGTGAACGGCAGGCATGAGTAC |
|  | REVERSE | TGGCGAGGATGGAGGAGGAATG |
| AKT1 | FORWARD | CAGGAGGAGGAGGAGATGGACTTC |
|  | REVERSE | CCCAGCAGCTTCAGGTACTCAAAC |
| AKT2 | FORWARD | AGGAGATGGAAGTGGCGGTCAG |
|  | REVERSE | GCAGGATCTTCATGGCGTAGTAGC |
| MTOR | FORWARD | CAACCAGCCAATCATTCGCATTCAG |
|  | REVERSE | ATGTCCGTTGCTGCCCATAAGTG |
| PGK1 | FORWARD | AGGTGCTCAACAACATGGAGATTGG |
|  | REVERSE | CAGGTATGCCAGAAGCCACAGTG |
| PGM1 | FORWARD | TTTGAATACAGCGACCCAGTGGATG |
|  | REVERSE | ATCTTGGCAACGTCCTTCTCATAGC |
| HK1 | FORWARD | CTGGACCGTCTGAATGTGACTGTG |
|  | REVERSE | CTGCTTGCCTCTGTGCGTAACC |
| PFKM | FORWARD | TCACAGATGAGGAGGCTACGAAGTC |
|  | REVERSE | GGTCATATCGGTGCCACAGAAGTC |
| PFKL | FORWARD | GCAAGGCATGAACGCTGCT |
|  | REVERSE | CCTCGTAGATGAGGAAGACTTTGGC |
| LDHB | FORWARD | ACTGTAGTGGGTGTTGGACAAGTTG |
|  | REVERSE | ACAAGAGCAAGTTCATCAGCCAGAG |
| IDH1 | FORWARD | GATGGCAAGACAGTAGAAGCAGAGG |
|  | REVERSE | ATGGAAGCAATGGGATTGGTGGAC |
| IDH2 | FORWARD | GTGGAGACGGTGGAGAGTGGAG |
|  | REVERSE | GGTGTTCAGGAAGTGCTCGTTCAG |
| FH | FORWARD | TTCCTTTACAGAAAACTGCGTG |
|  | REVERSE | TATGAGGATTGAGAGCTGTCAC |
| PDHA1 | FORWARD | TACAGGATGATGCAGACTGTAC |
|  | REVERSE | CAAGTGACAGAAACCACGAATA |
| PDHA2 | FORWARD | CTGATTACTACAAGAGGGGCAA |
|  | REVERSE | CAAATTTTGTTGCCTCACGAAC |
| PDHB | FORWARD | GACACTCCCATATCAGAGATGG |
|  | REVERSE | CTTGGCAGCTGAGTTTATAACC |
| ACO2 | FORWARD | TCAAGCTATGAAGATATGGGGC |
|  | REVERSE | GATCCCTCAAGATCTGTGCATA |
| GSS | FORWARD | CTCTTTGACATCCACAAGCAAG |
|  | REVERSE | AACATGTAGTCTGAGCGATTCA |
| GCLC | FORWARD | TGTCCGAGTTCAATACAGTTGA |
|  | REVERSE | ACAGCCTAATCTGGGAAATGAA |
| GLS | FORWARD | CACTCAAATCTACAGGATTGCG |
|  | REVERSE | CCAGACTGCTTTTTAGCACTTT |
| GAD1 | FORWARD | CTTCTTGCAAAGGACCAACAG |
|  | REVERSE | GTTTTCACAGGAAAGCAGGTTC |
| GAD2 | FORWARD | TCCAAAGTGGATGTCAACTACG |
|  | REVERSE | TCATAACATCTTGCAGAAACGC |
| GLUD1 | FORWARD | GACGACCCCAACTTCTTCAAG |
|  | REVERSE | TCCTCCACCAGCTTGTCCT |
| CPT1α | FORWARD | GATTTCCATTCCTTCCCATTCG |
|  | REVERSE | CTCGTATGTGAGGCAAAACTTG |
| CPT1β | FORWARD | AGAAGCACCAGAATATGTACCG |
|  | REVERSE | GAGAGCTGACTCCTAGGTACTT |
| CPT2 | FORWARD | GCCTAGATGACTTCCCCATTAA |
|  | REVERSE | AAAGGATTTATCAAACCAGCGG |
| ACADS | FORWARD | CAGTTACACACCATCTACCAGT |
|  | REVERSE | GCTGGGAAGAGATGTTCCTTAT |
| ACADL | FORWARD | GCATGGCGAAATATTGGGCATCTG |
|  | REVERSE | GGCTGAACTCTGGCATCCACATAAG |
| ACADVL | FORWARD | CAGCCTGGAAGGTGACAGATGAATG |
|  | REVERSE | AGAGCCACAAACAGCCGAAGAATG |
| ACADM | FORWARD | ATCGACAACGTGAACCAGGATTAGG |
|  | REVERSE | GCCACTGGGATGATTTCCTCTCTG |
| PPARα | FORWARD | TCGGCGAGGATAGTTCTGGAAGC |
|  | REVERSE | ACCACAGGATAAGTCACCGAGGAG |
| HK2 | FORWARD | GCAGGATGATTGCCTCGCAT |
|  | REVERSE | ACTGGTCAACCTTCTGCACTT |
| LDHA | FORWARD | AGGTGATCAAACTCAAAGGCTA |
|  | REVERSE | CCCAAAATGCAAGGAACACTAA |
| PKM | FORWARD | TGCCGCCTGGACATTGATTCAC |
|  | REVERSE | AGTTCAGACGAGCCACATTCATTCC |
| ENO1 | FORWARD | GTACCGCCACATCGCTGACTTG |
|  | REVERSE | GAACCGCCATTGATGACATTGAACG |
| GLUT1 | FORWARD | GATGAAGGAAGAGAGTCGGCAGATG |
|  | REVERSE | CAGCACCACAGCGATGAGGATG |
| GLUT3 | FORWARD | TTCAATGCTGATTGTCAACCTG |
|  | REVERSE | GCATTTCAACCGACTTAGCTAC |
| GLUT4 | FORWARD | CTGAAGGATGAGAAGCGGAAG |
|  | REVERSE | TCGAAGATGCTGGTCGAATAAT |
| MCT1 | FORWARD | GGCCCTGTTGAGAGAAATC |
|  | REVERSE | CAACTGCTGGTGGCATT |
| MCT4 | FORWARD | TCTGGATGGGTTGCTGA |
|  | REVERSE | TGGAAATGTGGTGGCTAAA |
| P16 | FORWARD | AGGCCGATCCAGGTCATGATGA |
|  | REVERSE | ACCACCAGCGTGTCCAGGAA |
| P21 | FORWARD | GATGGAACTTCGACTTTGTCAC |
|  | REVERSE | GTCCACATGGTCTTCCTCTG |
| P27 | FORWARD | CTAACTCTGAGGACACGCATTT |
|  | REVERSE | TTGAGTAGAAGAATCGTCGGTT |
| P53 | FORWARD | GCCGCAGTCAGATCCTAGC |
|  | REVERSE | CGTTGTTTTCAGGAAGTAGTTTCCA |
| P57 | FORWARD | CTGAACGCCGAGGACCAGAA |
|  | REVERSE | TCCACTTCGGTCCACTGCAG |
| PCNA | FORWARD | TAATTTCCTGTGCAAAAGACGG |
|  | REVERSE | AAGAAGTTCAGGTACCTCAGTG |
| Ki-67 | FORWARD | CAGACATCAGGAGAGACTACAC |
|  | REVERSE | GTTAGACTTGCTGCTGAGTCTA |
| cyclin A1 | FORWARD | GATGCTTGTCAGATACTCACCA |
|  | REVERSE | GTAGATGTCAAACCCTTGCTTG |
| cyclin A2 | FORWARD | AGAAACAGCCAGACATCACTAA |
|  | REVERSE | TTCAAACTTTGAGGCTAACAGC |
| cyclin B1 | FORWARD | GACTTTGCTTTTGTGACTGACA |
|  | REVERSE | CCCAGACCAAAGTTTAAAGCTC |
| cyclin B2 | FORWARD | GAGATATAAATGGACGCATGCG |
|  | REVERSE | AAATCGATCCATAATGCCAACG |
| cyclin C | FORWARD | CCTACATGTAGCCTGTGTTGTA |
|  | REVERSE | GATTGGCTGTAGCTAGAGTTCT |
| cyclin D1 | FORWARD | GTCCTACTTCAAATGTGTGCAG |
|  | REVERSE | GGGATGGTCTCCTTCATCTTAG |
| cyclin D2 | FORWARD | TTTAAGTTTGCCATGTACCCAC |
|  | REVERSE | ACGTCTGTGTTGGTGATCTTAG |
| cyclin D3 | FORWARD | CTTACTGGATGCTGGAGGTATG |
|  | REVERSE | GTAGCGATCCAGGTAGTTCATG |
| cyclin E1 | FORWARD | TTGTGTCCTGGCTGAATGTATA |
|  | REVERSE | AAGGAAATTCAAGGCAGTCAAC |
| cyclin E2 | FORWARD | AGCTCAGCTTTTAGATCTGTGT |
|  | REVERSE | TTCTGAAATACTGTCCCACTCC |
| CDK1 | FORWARD | CACAAAACTACAGGTCAAGTGG |
|  | REVERSE | GAGAAATTTCCCGAATTGCAGT |
| CDK2 | FORWARD | CCTGGGCTGCAAATATTATTCC |
|  | REVERSE | TGGCTTGTAATCAGGCATAGAA |
| CDK4 | FORWARD | TTTTGAGCATCCCAATGTTGTC |
|  | REVERSE | TCGACGAAACATCTCTTGATCT |
| CDK6 | FORWARD | CGAACAGACAGAGAAACCAAAC |
|  | REVERSE | CTCGGTGTGAATGAAGAAAGTC |
| PGC1α | FORWARD | CAGAGAGTATGAGAAGCGAGAG |
|  | REVERSE | AGCATCACAGGTATAACGGTAG |
| PGC1β | FORWARD | CTTCCTCAACTATCTCGCTGACACG |
|  | REVERSE | GGGAGAGGTCAAGTTCTGGAAAGTC |
| PRC | FORWARD | AACTACGGCTTCGTCACTTATC |
|  | REVERSE | AGATCAGAATAGCTCCTCTTGC |
| SOD1 | FORWARD | ATCCTCTATCCAGAAAACACGG |
|  | REVERSE | GCGTTTCCTGTCTTTGTACTTT |
| SOD2 | FORWARD | CGCCCTGGAACCTCACATCAAC |
|  | REVERSE | AACGCCTCCTGGTACTTCTCCTC |
| CAT | FORWARD | GAGCACAGCATCCAATATTCTG |
|  | REVERSE | CTCATTCAGCACGTTCACATAG |
| GPX1 | FORWARD | GTTGCCTGGAACTTTGAGAAG |
|  | REVERSE | CTCGATGTCAATGGTCTGGAAG |
| GPX3 | FORWARD | CATAAGTGGCACCATTTACGAG |
|  | REVERSE | AAGGATCTCTGAGTTCTCTCCT |
| GPX4 | FORWARD | ATGGTTAACCTGGACAAGTACC |
|  | REVERSE | GACGAGCTGAGTGTAGTTTACT |
| β-actin | FORWARD | CATGTACGTTGCTATCCAGGC |
|  | REVERSE | CTCCTTAATGTCACGCACGAT |
| TFAM | FORWARD | ACAGGATGATGACTATGGAA |
|  | REVERSE | CAACTCTGAATACAATGTGAATT |
| NRF1 | FORWARD | GCTGATGAAGACTCGCCTTCT |
|  | REVERSE | TACATGAGGCCGTTTCCGTTT |
| NRF2 | FORWARD | GAGAGCCCAGTCTTCATTGC |
|  | REVERSE | TGCTCAATGTCCTGTTGCAT |
| SDHB | FORWARD | CACTCTAGCTTGCACCCGAA |
|  | REVERSE | CGTAGAGCCCGTCCAGTTTC |
| ATP5A1 | FORWARD | ACTGGGCGTGTCTTAAGTATTG |
|  | REVERSE | CACATCAACTACGGGAACCA |
| COXII | FORWARD | ATTTACAGGGTAACTGCTTAGG |
|  | REVERSE | CCCCCTCCTTGTTTCTTGGA |
| NDUFS3 | FORWARD | TTGGAGAGTATGTGGCTGAAAT |
|  | REVERSE | GAGATTTGAACTGTGCATTGGT |
| NDUFB8 | FORWARD | CTCCTTGTTGGGCTTATCACA |
|  | REVERSE | GCCCACTCTAGAGGAGCTGA |
| UQCRC2 | FORWARD | GTTTGTTCATTAAAGCAGGCAGTAG |
|  | REVERSE | CTATTGGGCACCTTAACTTCGT |
| ND1 | FORWARD | CACACTAGCAGAGACCAACCGAAC |
|  | REVERSE | CGGCTATGAAGAATAGGGCGAAGG |
| H19 | FORWARD | AGGAATCGGCTCTGGAAGGTGAG |
|  | REVERSE | TTTGATGTTGGGCTGATGAGGTCTG |

**Table S3.** Primary antibodies

| Antibodies | Source | Identifier |
| --- | --- | --- |
| PGC1α | Affinity | AF5935 |
| β actin | Huabio | EM21002 |
| ATP5A1 | Huabio | ET1703-53 |
| SDHB | Huabio | ET1706-30 |
| MTCO2 | Huabio | ET1610-72 |
| NDUFS3 | Huabio | ET1706-07 |
| UQCRC2 | Huabio | ER1803-08 |
| AMPK | Huabio | ET1608-40 |
| p-AMPK | Huabio | ET1612-72 |
| mtTFA | Huabio | ET1705-64 |
| NRF1 | Huabio | ET1705-86 |
| Nrf2 | Huabio | ET1706-41 |
| p16INK4A | Huabio | ET1608-62 |
| anti-Mouse IgGs | Huabio | HA1006 |
| anti-Rabbit IgGs | Huabio | HA1001 |
| Bax | CST | 41162 |
| Bcl-2 | CST | 15071 |
| Caspase-3 | CST | 9662 |
| p-AKT | CST | 9271 |
| PCNA | Santa | sc-56 |
| LDHA | Santa | sc-137243 |
| p-PI3K | Zenbio | R22768 |
| PI3K | Zenbio | 380934 |
| AKT | Zenbio | R23412 |
