# Supplementary figures and images for: Knockdown of PGC1α suppresses dysplastic oral keratinocytes proliferation through reprogramming energy metabolism
Source: Int J Oral Sci. 2023 Sep 4;15:37. doi: 10.1038/s41368-023-00242-3 (PMC10475463; doi:10.1038/s41368-023-00242-3)

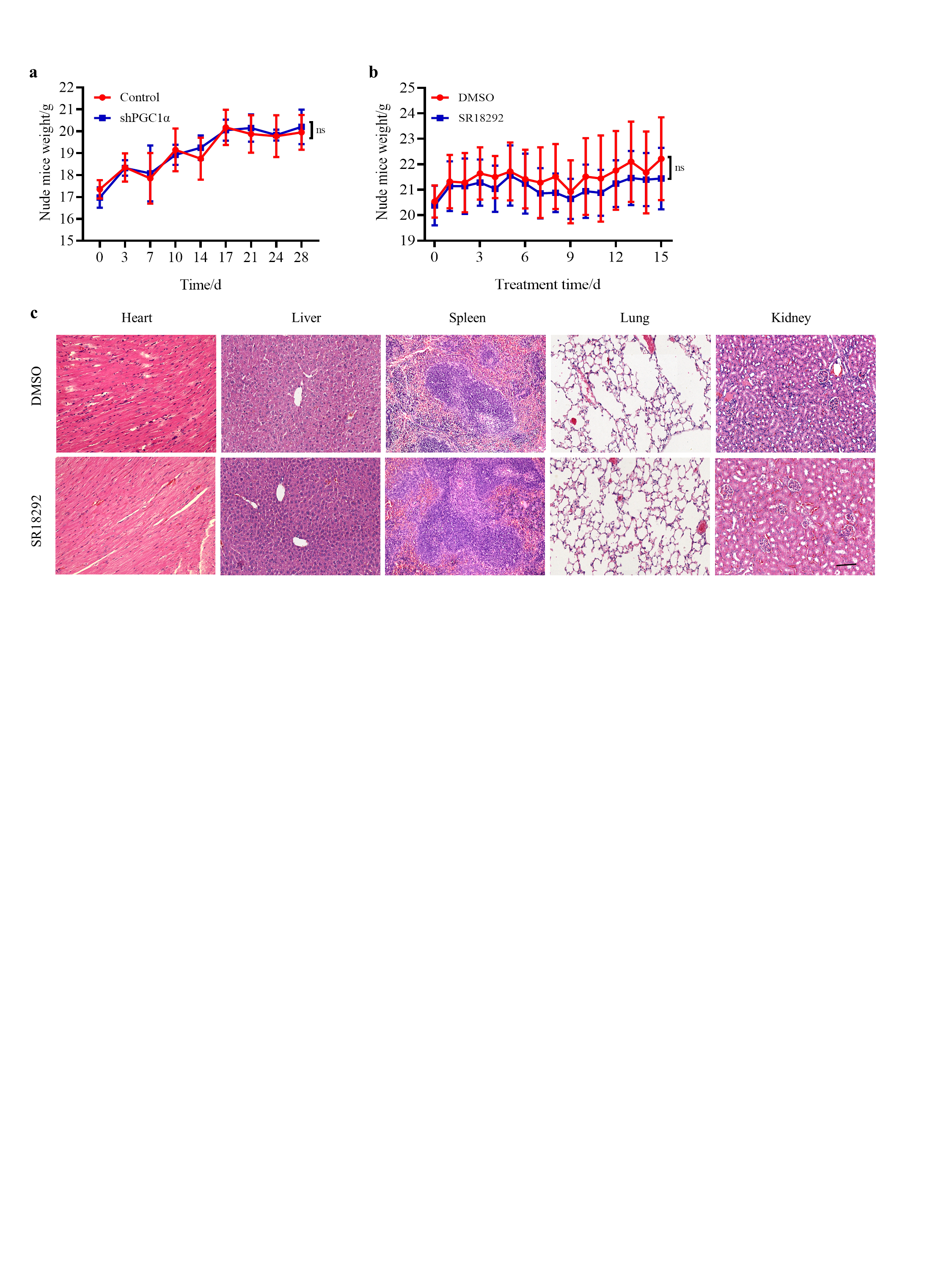

Supplement: Supplementary file 2 — Fig. S1 [file 41368_2023_242_MOESM2_ESM.tif]

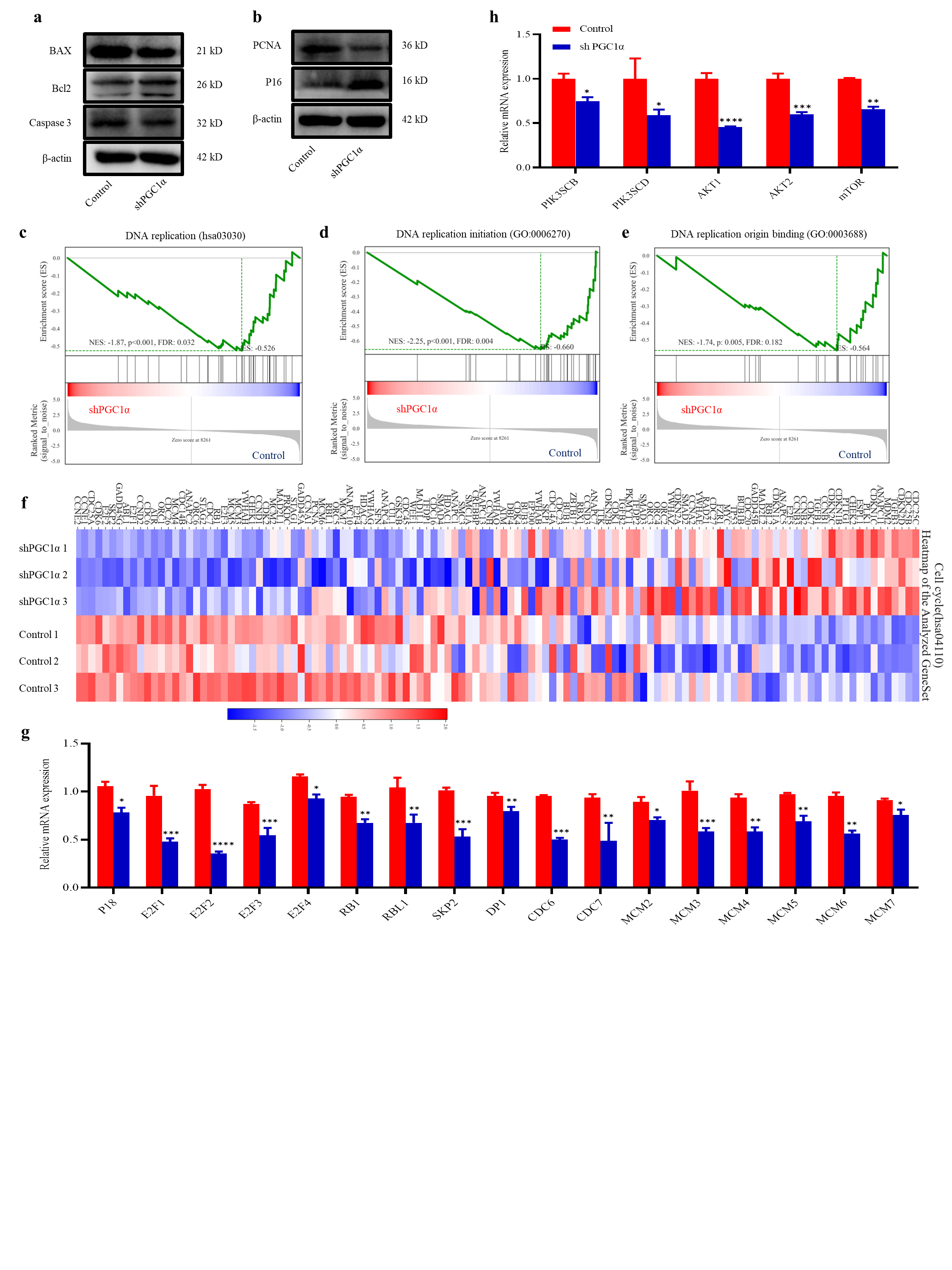

Supplement: Supplementary file 3 — Fig. S2 [file 41368_2023_242_MOESM3_ESM.tif]

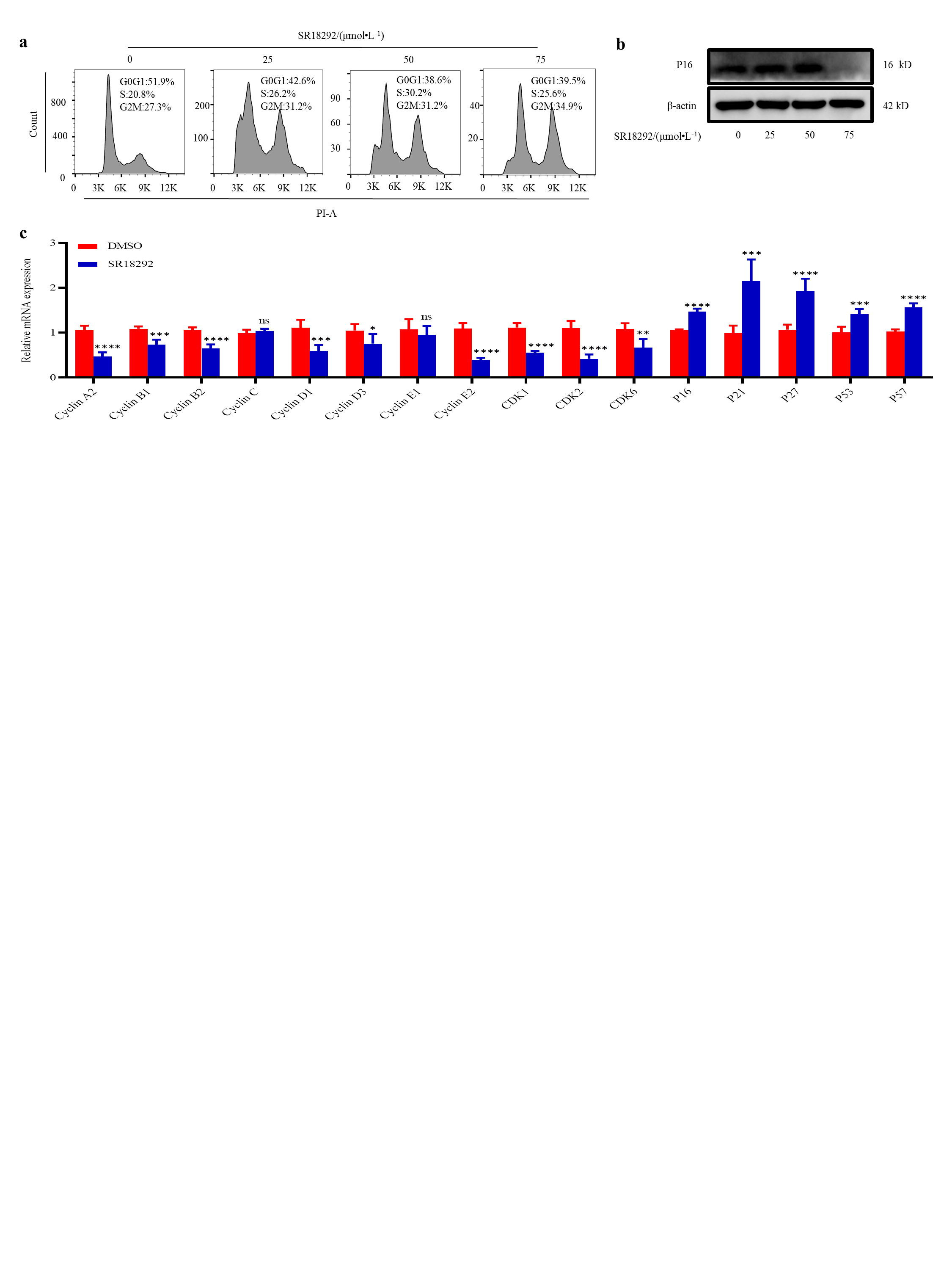

Supplement: Supplementary file 4 — Fig. S3 [file 41368_2023_242_MOESM4_ESM.tif]

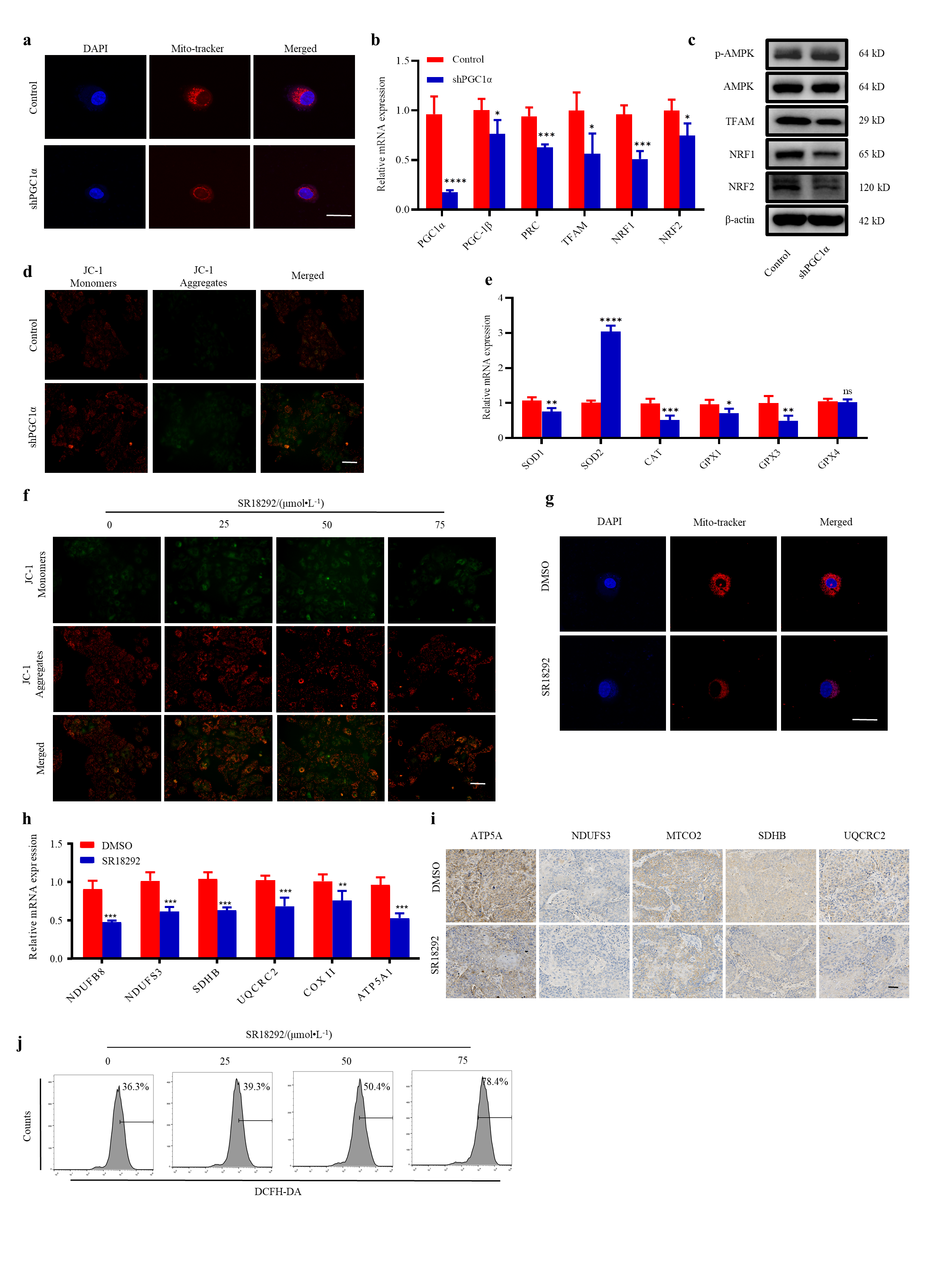

Supplement: Supplementary file 5 — Fig. S4 [file 41368_2023_242_MOESM5_ESM.tif]
